# Prevalence of HPV High-Risk Genotypes in Three Cohorts of Women in Ouagadougou (Burkina Faso)

**DOI:** 10.4084/MJHID.2013.059

**Published:** 2013-09-02

**Authors:** Theodora M. Zohoncon, Cyrille Bisseye, Florencia W. Djigma, Albert T. Yonli, Tegwinde R. Compaore, Tani Sagna, Djeneba Ouermi, Charlemagne M.R. Ouédraogo, Virginio Pietra, Jean-Baptiste Nikiéma, Simon A. Akpona, Jacques Simpore

**Affiliations:** 1 “Pietro Annigoni” Centre for Bio-molecular Research CERBA/LABIOGENE, University of Ouagadougou, 01 BP 364 Ouagadougou 01, Burkina Faso; 2 Saint-Camille Medical Center, 09 BP 444 Ouagadougou 09, Burkina Faso; 3 University of Parakou, PO Box 123, Republic of Benin; 4 Department of Biology, University of Science and Technique of Masuku (USTM); BP 943 Franceville, Gabon

## Abstract

The development of cervical cancer is associated with high-risk Human papilloma viruses (HPV-HR). In sub-Saharan Africa cervical cancer is the most common cancer among women and the leading cause of death attributed to malignant tumors. This study aims to identify HPV genotypes within the 30′S and 50′S HPV families found in two previous studies from our laboratory, and to determine the prevalence of twelve HPV-HR genotypes in a population of women in Ouagadougou. The twelve HPV-HR genotypes were determined by real-time multiplex PCR, in 180 samples from the general population and among a group of HIV-1 infected women.

The most common genotypes found were HPV-35 (29.4%) and HPV-31 (26.1%) of the 30′S family, and HPV-52 (29.4%) and HPV-58 (20.6%) of the 50′S family. Multiple infections of HPV-HR were observed in 78.03% of infected women.

The frequencies of HPV genotypes from the 30′S and 50′S families were higher, while the genotypes HPV-16 and18 were lower among the women in our study.

## Introduction

Human papilloma viruses (HPV) are responsible for cervical cancer (CC), genital warts and verruca.^1^ They are the most common viruses in sexually transmitted infections.^1^–^3^ Their involvement in the development of cervical cancer is well established.^4^ In sub-Saharan Africa, CC is the most common cancer in women and the leading cause of mortality due to cancer.^5^ The global incidence of cervical cancer is 493,000 new cases each year, and 80% of women affected live in developing countries.^6^ CC and other HPV related diseases are a real public health problem worldwide.^7^, ^8^ Human immunodeficiency virus (HIV) and HPV co-infection is a risk factor for HPV’s infection persistence in Africa.^6^ In 2001, among women between the age of 15 and 49, HIV prevalence was of 3.5% in Ouagadougou (Burkina Faso).^9^ Among High Risk Human Papillomaviruses (HR-HPV), only types 16 and 18 are covered by the available HPV vaccines (Gardasil ® and Cervarix ®). Two previous studies from our laboratory carried out in Ouagadougou in 2011 by Djigma et al., and Ouedraogo et al. showed a high prevalence of HPV-HR from the 30′S (31, 33, 35, 39) and 50′S (51, 52, 56, 58, 59) families.^10^, ^11^ However, the method of HPV genotyping (Use of polymerase chain reaction and hybridization assays) did not distinguish the different genotypes within 30′S and 50′S HPV families. The objective of this study was, firstly to determine the different genotypes within 30′S and 50′S HPV families detected in the two previous studies cited above and secondly to diagnose by real-time PCR, twelve HPV-HR genotypes in a cohort of women attending gynecological consultation in Ouagadougou.

## Material and Methods

### Patients

One hundred and eighty (180) women divided into three (3) groups were included in this study.

Cohort 1 consisted of 63 women of unknown HPV status, recruited from November 2012 to January 2013. Cohorts 2 and 3 were formed by 34 women positive for 30′S and 50′S HPV^11^ and 83 HIV-1-seropositive women positive for HPV-30′S and/or HPV50′S.^10^ Each woman completed a questionnaire to help determine her socio-economic, occupational and behavioral habits.

All women who could write signed a consent forms to participate to the study.

### Sample collection

Samples were collected from the endocervical region with a sterile cotton swab. The collected samples were stored in transport medium (Sacace biotechnologies) and frozen at − 20° C.

### Extraction of viral DNA and genotyping of HPV-HR

DNA extraction was performed using the DNA-Sorb-A kit (Sacace Biotechnologies, Como, Italy) following the manufacturer’s instructions. HPV-HR genotyping was performed by real time PCR using the HPV High Risk Typing Real-TM kit (Sacace Biotechnologies) in the SaCycler-96 Real Time PCR machine (Sacace Biotechnologies).

The HPV High Risk Typing Real-TM kit is based on two major processes: isolation of DNA from specimens, and multiplex Real Time amplification of 4 tubes for each sample. Each tube contains primers directed against regions of three HPV types and the β-globin gene used as Internal Control.

PCR conditions were as follows : 1 cycle of 95°C for 15 minutes ; 5 cycles of 95°C for 05s, 60°C for 20s and 72°C for 15s; and 40 cycles of 95°C for 05s, 60°C for 30s and 72°C for 15s.

### Statistical analysis

Data were analyzed using SPSS 17 .0 and Epi Info 3.5.1 softwares. The Chi-square test was used for comparisons. A difference was significant for p <0.05. The confidence interval (CI) and odd ratios (OR) were calculated with Epi Info 6.

## Results

### Socio-demographic characteristics of women

The average age of women in cohorts 1 and 2 were respectively 33.2 ± 8.3 years (21 – 51) and 31.5 ± 10.3 years (19 – 60). Among the HIV-1 seropositive women, the mean age was 33.7 ± 6.2 years (20–53). The majority of the HPV-HR infected women was married and had a low level of education. Almost all of the women in the study had only one sexual partner. Table 1 shows the demographic characteristics of the women included in the study.

### Prevalence of HR-HPV genotypes

Among all the 180 HPV-HR genotypes found, the most frequent were HPV-35 (29.4%) and HPV-52 (29.4%) followed by HPV-31 (26.1%), HPV-18 (25. 0%), HPV-58 (20.6%), HPV-56 (18.9%), HPV-51 (14.4%), HPV-59 (13.9%), HPV-45 (12.8%) and HPV-33 (11.1%). The least frequent genotypes were HPV-16 (7.2%) and HPV-39 (6.7%). Among the HIV-1 seropositive women, the most represented genotype was HPV-35 with a prevalence of 49.4% while HPV-39 (9.6%) was the least common genotype. In women from cohort 1, 30.20% (19/63) were infected with at least one HPV-HR. HPV-52 (15. 9%) was more prevalent while HPV-16 with a frequency of 1.6% was less frequent (Table 2). The cumulative prevalence of HPV-30′S and HPV-50′S was 43.0% in cohort 1 while the most common genotypes in cohort 2 was HPV-52 (35.3%) followed by HPV-58 (32.4%), HPV-35 (26.5%) and HPV-31 (20.6%). Table 3 shows the frequencies of the twelve HPV-HR genotypes among HIV positive women and women of unknown HIV status.

### Number of HPV-HR genotypes in infected women

The number of HPV-HR genotypes in women ranged from 1 to 7 in HIV-1 seropositive women, and 1 to 5 in women from cohorts 1 and 2 (general population). The average number of genotype HPV-HR was significantly higher in HIV-1 positive women (3.5 ± 1.52) compared to women of the general population (2.1 ± 1.17) (p <0.001). In addition the majority of HIV-1 positive women (74.1%) had at least 3 HPV-HR genotypes, while the majority of women of the general population (66.7%) had no more than 2 HPV-HR genotypes (Table 4).

HPV-HR multiple infections were observed in 78.03% of the women of the study; but were 90.1% in HIV-1 infected women.

## Discussion

This study first aim was to diagnose individually by real-time PCR genotypes HPV-HR 30′S and 50′S detected in two previous studies from our laboratory and the second aim was to genotype 12 HPV-HR in a cohort of women in Ouagadougou.

The prevalence of 30.2% of HPV-HR infection observed in this study (cohort 1) was comparable to that of 27.9% reported in Colombia.^12^ It was less than the prevalence of 72.6% reported in Burkina Faso^11^, but more than the prevalence of 7.9% and 15.6% respectively reported in China^13^ and Nigeria.^14^ This could be explained by the fact that the majority of the women in our study had one sexual partner. Indeed, it has been reported that having many sexual partners is associated with a high prevalence of HPV infection.^12^

In cohort 1 of this study (cohort 1) the cumulative prevalence of HPV-30′S and HPV-50′S families was 43.0%. We also found that the HPV-52 genotype was the most frequent both in our study population as well as in cohort 2 the (study by Ouedraogo et al, 2011). However, HPV-16 was the least common genotype observed in women. Our findings support those of Chen et al. who observed a high prevalence of HPV-52 (33.4%) and HPV-58 (15.93%) and a relatively low prevalence of HPV-16 (20.95%) and HPV-18 (8.36%) among women in China.^13^ HPV-HR genotypes (HPV-35, HPV-31, HPV-18 and HPV-33) infections prevalence was significantly higher among HIV-seropositive women than among women in the general population. HPV-35 was the most prevalent in seropositive women with a frequency of 49.4%. Our results are in agreement with previous studies which reported that the prevalence of HPV infection is higher among HIV-seropositive women.^15^–^22^ In Tanzania and South Africa Dols et al.^23^ reported that the most frequent HPV-HR in HIV positive women were HPV-52 (30%) and HPV-16 (26%), there was no regional difference in the prevalence of HPV-18 and HPV-35.

Indeed, HIV increases the risk of developing cervical intra-epithelial neoplasia (CIN) and cervical cancer of the uterus.^24^ A recent study showed that the majority of HPV-HR genotypes found in this study among HIV-positive women had been detected in women with invasive cervical cancer, CIN2 and CIN3.^25^ We examined the carriage of HR-HPV genotypes among women of the three cohorts and found a significantly higher number of HPV-HR genotypes in HIV-positive women compared with those of the general population. Most HIV-positive women (74.1%) had at least 3 HPV-HR genotypes, while the majority of women in the general population (66.7%) had a maximum of 2 HPV-HR genotypes. Our results corroborate those found in South Africa which reported that HIV-positive women had an average of 2.4 HPV types per sample while HIV-negative women had an average of 0.7 HPV genotypes.^26^ Multiple HPV-HR infection was observed in 78.03% of infected women. 90.1% of the HIV-1 infected women had several HPV-HR infections.

The major limitation of this report is the sample size which is not representative of the female population of Ouagadougou. However, this pilot study has an epidemiological and clinical importance because it shows the prevalence of HR-HPV genotypes in Burkina Faso is different from other regions of the world with a preponderance of HPV-35, HPV-52, HPV-31 and HPV-18 genotypes. This study can have a real impact on the choice of immunization policy of the country as the existing HPV vaccines only cover HPV-16, HPV-18, HPV-6 and HPV-11 genotypes. The development of HPV vaccines covering the of HPV genotypes 30′S and 50′S families should be considered to protect all women from getting infected with these viruses.

## Conclusion

We showed in this study a predominance of genotypes HPV-35, HPV-52, HPV-31 and HPV-18, respectively in HIV-1 seropositive women and in those of the two cohorts studied from the general population. A low prevalence of HPV-16 genotypes was also found. A large-scale study is needed to determine the epidemiology of HPV in the population in Burkina Faso.

## Figures and Tables

**Table 1:** Socio-demographic, sexual and behavioral characteristics of women infected with high-risk HPV

	Women of cohort 1 HPV positive n (%)	Women of cohort 2 HPV positive n (%)	HIV seropositive women HPV positive n (%)
**Age**			
≤ 30 years	8 (42.1)	21 (61.8)	27 (32.5)
>30 years	11 (57.9)	13 (38.2)	56 (67.5)

**Age at the time of the first sexual intercourse**			
< 20 years	11 (57.9)	25 (73.5)	50 (60.2)
≥ 20 years	8 (42.1)	9 (26.5)	19 (22.9)
Not mentioned	-	-	14 (16.9)

**Use of preservatives**			
yes	3 (15.8)	2 (5.9)	31 (37.3)
No	12 (63.2)	19 (55.9)	14 (16.9)
Sometimes	4 (21.0)	8 (23.5)	15 (18.1)
Notspecified	-	5 (14.7)	23 (27.7)

**Oral Contraception**			
Yes	1 (5.3)	5 (14.7)	61 (73.5)
No	18 (94.7)	29 (85.3)	21 (25.3)
Not specified	-	-	1 (1.2)

**Number of sexual partners**			
None	0 (0.0)	0 (0.0)	0 (0.0)
1	19 (100)	28 (82.4)	52 (62.7)
≥ 2	0 (0.0)	1 (2.9)	0 (0.0)
	-	5 (14.7)	31 (37.3)

**Matrimonial Situation**			
Single	6 (31.6)	9 (26.5)	12 (14.5)
Married	11 (57.9)	24 (70.6)	43 (51.8)
Widow	2 (10.5)	1 (2.9)	18 (21.7)
Divorced	-	-	10 (12.0)

**Level of education**			
None	10 (52.6)	12 (35.3)	28 (34.1)
Primary	4 (21.1)	5 (14.7)	29 (35.4)
Secondary	2 (10.5)	10 (29.4)	22 (26.9)
Superior	3 (15.8)	7 (20.6)	3 (3.7)

**Tobacco Consumption**			
Yes	0 (0.0)	0 (0.0)	0 (0.0)
No	19 (100)	34 (100)	83 (100)

**HIV Status**			
unknown	7 (36.8)	-	0 (0.0)
Negative	9 (47.4)		0 (0.0)
Positive	3 (15.8)		83 (100)

**Table 2:** Prevalence of the twelve HPV-HR genotypes among the 63 women of unknown HPV status

HPV Genotypes	Prevalence n (%)	Confidence Interval 95%
HPV-16	1 (1.6)	0.1–9.7
HPV-18	4 (6.3)	2.1–16.3
HPV-31	4 (6.3)	2.1–16.3
HPV-33	0 (0.0)	0.0–7.2
HPV-35	3 (4.8)	1.2–14.2
HPV-39	2 (3.2)	0.6–12.0
HPV-45	3 (4.8)	1.2–14.2
HPV-51	3 (4.8)	1.2–14.2
HPV-52	10 (15.9)	8.3–27.7
HPV-56	2 (3.2)	0.6–12.0
HPV-58	2 (3.2)	0.6–12.0
HPV-59	1 (1.6)	0.1–9.7
**Total HPV-HR**	**19 (30,2)**	**19.6–43.2**

**Table 3:** Frequency of the twelve HPV-HR genotypes among HIV+ women and women of HIV unknown status

	HIV+ N = 83		HIV unknown status N = 53			

HPV Genotypes	Frequency n (%)	Confidence Interval 95%	Frequency n (%)	Confidence Interval 95%	Odds Risk (Confidence Interval 95%)	*p values*
HPV-16	10 (12.0)	6.2–21.5	3 (5.7)	1.5–16.6	2.3 (0.5–11.1)	NS
HPV-18	36 (43.4)	32.7–54.7	9 (17.0)	8.5–30.3	3.7 (1.5–9.5)	<0.01
HPV-31	36 (43.4)	32.7–54.7	11 (20.8)	11.3–34.5	2.9 (1.2–7.0)	<0.01
HPV-33	18 (21.7)	13.7–32.4	2 (3.8)	0.7–14.1	7.0 (1.5–46.3)	<0.01
HPV-35	41 (49.4)	38.3–60.5	12 (22.6)	12.7–36.6	3.3 (1.5–7.8)	<0.01
HPV-39	8 (9.6)	4.6–18.6	4 (7.5)	2.5–19.1	1.3 (0.3–5.5)	NS
HPV-45	15 (18.1)	10.8–28.4	8 (15.1)	7.2–28.1	1.2 (0.5–3.5)	NS
HPV-51	18 (21.7)	13.7–32.4	8 (15.1)	7.2–28.1	1.6 (0.6–4.3)	NS
HPV-52	31 (37.3)	27.2–48.7	22 (41.5)	28.4–55.8	0.8 (0.4–1.8)	NS
HPV-56	25 (30.1)	20.8–41.3	9 (17.8)	8.5–30.3	2.1 (0.8–5.4)	NS
HPV-58	24 (28.9)	19.7–40.1	13 (24.5)	14.2–38.6	1.3 (0.5–3.0)	NS
HPV-59	19 (22.9)	14.7–33.7	6 (11.3)	4.7–23.7	2.3 (0.8–7.1)	NS

**Table 4:** Number of women infected by HPV genotypes

Number of HPV genotypes identified per woman	Number of HIV-1 positive women		Number of women in cohort 1 and 2	

	Absolute Number	(%)	Absolute Number	(%)
1	8	9,9	21	41.2
2	13	16	13	25.5
3	24	29,6	10	19.6
4	16	19,8	5	9.8
5	12	14,8	2	3.9
6	5	6,2	0	0.0
7	3	3,7	0	0.0
Total	81	100	51	100
